# Directional Polarization‐Independent Double‐Zero‐Index Material in a Three‐Dimensional Photonic Crystal With a Fourfold Semi‐Dirac Point

**DOI:** 10.1002/nap2.70045

**Published:** 2026-03-20

**Authors:** Rui Dong, Ying Wu, Changqing Xu

**Affiliations:** ^1^ Key Laboratory of State Manipulation and Advanced Materials in Provincial Universities, School of Physics and Technology Nanjing Normal University Nanjing China; ^2^ Division of Computer, Electrical and Mathematical Science and Engineering King Abdullah University of Science and Technology (KAUST) Thuwal Saudi Arabia; ^3^ Physical Science and Engineering Division King Abdullah University of Science and Technology (KAUST) Thuwal Saudi Arabia

## Abstract

We propose a strategy to realize directional polarization‐independent double‐zero‐index materials within a three‐dimensional photonic crystal. In the transition between two nodal rings with opposite Zak phases, a fourfold semi‐Dirac point emerges at the Brillouin zone center. This point exhibits linear dispersions along one direction and quadratic dispersions along perpendicular directions in the momentum space. At the frequency of the semi‐Dirac point, both the effective permittivity and permeability of the photonic crystal simultaneously reach zero along the direction of linear dispersions, whereas only one of them becomes zero along the directions of quadratic dispersions. We investigate typical wave manipulation characteristics of this directional double‐zero‐index material, such as total transmission of circularly polarized electromagnetic waves and highly collimated directional emission. Our study clarifies the connection between topological phase transitions, band dispersions, and double‐zero‐index properties in artificial materials, offering a general method for achieving directional double‐zero‐index materials with potential applications in wave manipulation.

## Graphical abstract

We propose a strategy to realize directional polarization‐independent double‐zero‐index materials within a three‐dimensional photonic crystal which possesses a fourfold semi‐Dirac point. This point exhibits linear dispersions along one direction and quadratic dispersions along perpendicular directions in the momentum space. At the frequency of the semi‐Dirac point, both the effective permittivity and permeability of the photonic crystal simultaneously reach zero along the direction of linear dispersions.

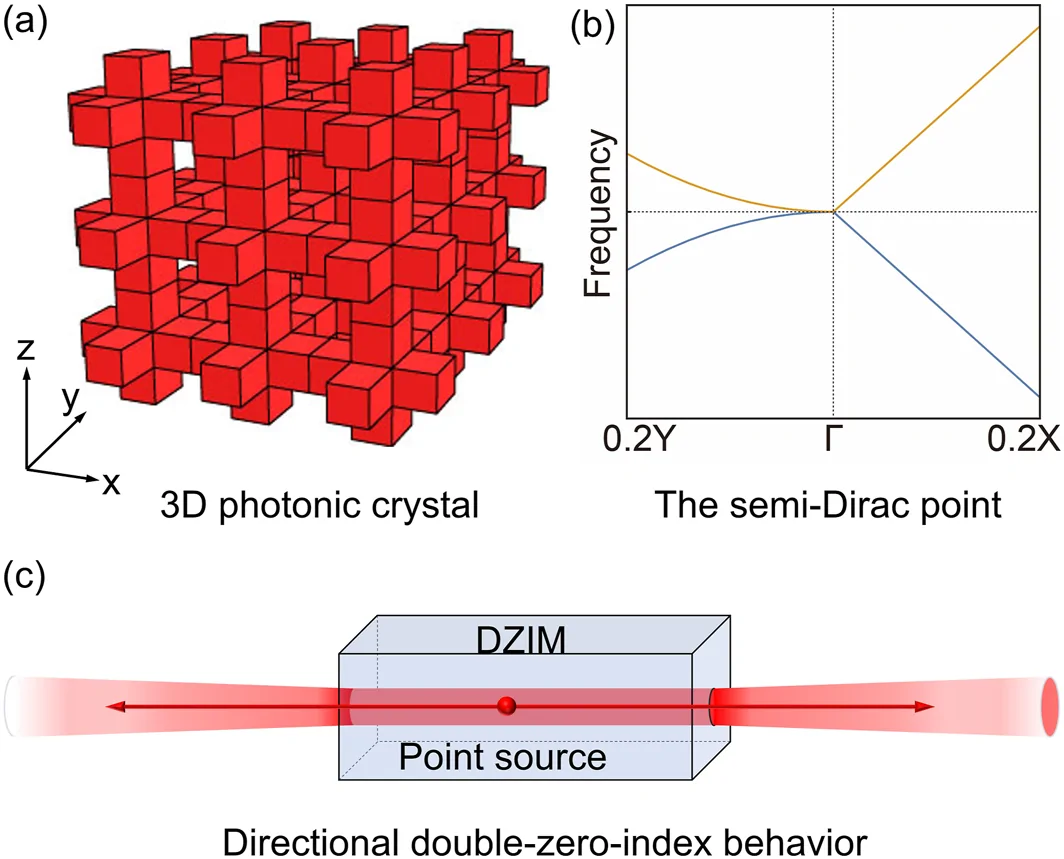

## 1 Introduction

Zero‐index materials (ZIMs) exhibit unique wave manipulation capabilities due to their vanishing constitutive parameters, which eliminate phase retardation during wave propagation. This fundamental characteristic enables diverse functionalities including wavefront engineering [1, 2, 3, 4, 5, 6], impurity cloaking [7, 8, 9, 10, 11], wave tunneling [12, 13, 14, 15, 16, 17], photonic doping [18, 19, 20, 21, 22, 23, 24, 25, 26], enhancement of radiance [27] and optical nonlinear effects [28], etc. ZIMs are generally categorized into two types: Single‐zero‐index materials (SZIMs) with either permittivity or permeability approaching zero, with inherent impedance usually mismatched with conventional materials [29, 30, 31], and double‐zero‐index materials (DZIMs) with both constitutive parameters approaching zero, possessing better matched impedance to the ambient environment [32, 33, 34, 35, 36]. In general, the SZIMs can maintain quantum entanglement over extended distances and time periods [37], whereas DZIM enables in‐plane wavefront engineering and directional emission [38, 39, 40, 41, 42, 43].

In two‐dimensional (2D) systems, DZIMs for in‐plane waves are typically realized through two well‐established approaches: photonic doping via engineering of impurities [18, 19, 20, 21, 22, 23, 24, 25, 26] and Dirac‐like conical dispersions formed by accidental degeneracy of eigenstates [38, 39, 40, 41, 42, 43]. However, in a three‐dimensional (3D) electromagnetic system, the photonic doping is no longer applicable due to the inherently vectorial nature of the 3D electromagnetic waves, introducing a counterintuitive “anti‐doping” effect [11] and imposes new challenges. The polarization of electromagnetic waves becomes fixed when they propagate through 3D DZIMs in a transverse electromagnetic waveguide [36]. Because of the metal losses in the visible to near‐infrared regime, it becomes challenging to emulate ideal boundary conditions, such as perfect electrical conductors or perfect magnetic conductors. These limitations highlight the need to develop a ZIM that can intrinsically restrict the propagation of electromagnetic waves, thereby eliminating the reliance on external boundary conditions [44].

To address this issue, we propose a design of directional DZIM using a 3D photonic crystal (PC) with a tetragonal lattice of dielectric meshes. By changing the geometric parameters of the PC, we achieve accidental degeneracy of electric and magnetic dipolar states at the Brillouin zone center, forming a fourfold semi‐Dirac point. Near this point, the dispersions exhibit a linear behavior along one direction and quadratic along the other two perpendicular directions, which can be understood from a tight‐binding model. Different from its 2D counterparts, which is the annihilation of two Dirac points with opposite Berry connections [45, 46], the semi‐Dirac point in 3D space represents a topological transition between two nodal rings with opposite Zak phases [47, 48, 49, 50]. At the frequency of the semi‐Dirac point, the PC can be effectively regarded as a DZIM along the direction of linear dispersions and a SZIM along the directions of quadratic dispersions. The anisotropic zero‐index property results in unique wave manipulation functionalities, such as directional total transmission for circularly polarized electromagnetic waves and highly collimated directional emission.

## 2 3D Semi‐Dirac Point and Nodal Rings

Figure 1a shows the unit cell of the 3D PC, which is composed of a tetragonal lattice of dielectric meshes with relative permittivity ε=9. The lattice constants along the x,y,z‐directions are set as $a_{x}=1.25a$ and $a_{y}=a_{z}=a$, respectively. The meshes are aligned with the coordinate axes, intersecting at the center of the unit cell. Along different directions, the cross‐sections of the meshes have side lengths of $b_{i}=La_{i}$ (i=x,y,z), where L is a dimensionless number. We use COMSOL Multiphysics, a commercial package based on finite‐element, to calculate the band structure of this PC along the high‐symmetry lines of the first Brillouin zone and plot the results in Figure 1c with dimensionless frequency 2πc/a, where *c* is the speed of light in vacuum. At the Γ point, we observe an accidental degeneracy of electric dipolar‐*y*,*z* states (shown in Figure 1d,e, labeled as $e_{y}$ and $e_{z}$, respectively) and magnetic dipolar‐*y*,*z* states (shown in Figure 1f,g, labeled as $m_{y}$ and $m_{z}$, respectively) at frequency $\omega_{s}=0.475\left(2\pi c/a\right)$. Semi‐Dirac dispersions are observed near this degenerate point, featuring linear bands along the ΓX direction and quadratic bands along the ΓY direction (as well as ΓZ direction because of lattice symmetry).

**FIGURE 1:**
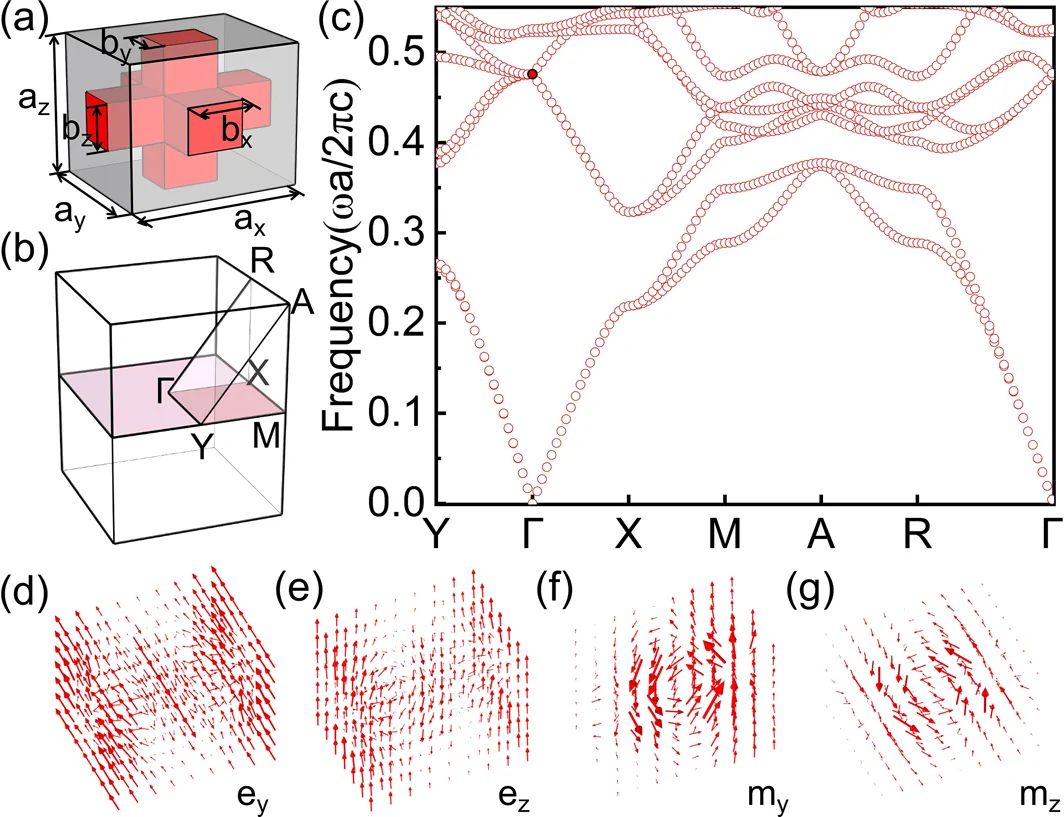
3D PC with a semi‐Dirac point at the center of Brillouin zone. (a) The unit cell of the PC. The side lengths of the dielectric mesh are set as $b_{i}=La_{i}$ (i=x,y,z). (b) The scheme of Brillouin zone with high symmetry points. (c) Band structure of the PC when L=0.327, exhibiting a fourfold semi‐Dirac point (solid circle) at the Γ point. (d–g) The electric field arrow maps of the (d) electric dipolar‐*y* states, (e) electric dipolar‐*z* states, (f) magnetic dipolar‐*y* states, and (g) magnetic dipolar‐*z* states.

From the Maxwell equation, the eigenvalue problem of this PC is given by the following equation:

(1) $$\hat{H}H_{k}\left(r\right)=\frac{\omega_{k}^{2}}{c^{2}}H_{k}\left(r\right),$$

where $\hat{H}=\nabla \times \left(\frac{1}{\varepsilon \left(r\right)}\nabla \times \right)$ is the Hamiltonian, $\omega_{k}$ is the eigenfrequency corresponding to wave vector k, $H_{k}\left(r\right)$ and ε(r) are the spatial distribution of magnetic field at k and the spatial distribution of permittivity, respectively. Near the semi‐Dirac point, the tight‐binding Hamiltonian can be written in the basis of electric dipolar‐*y*,*z* states and magnetic dipolar‐*y*,*z* states as follows:

(2) $$\hat{H}=\left(\begin{array}{cccc} E+q{\left|k\right|}^{2}+\Delta & 0 & 0 & ipk_{x}\\ 0 & E-q{\left|k\right|}^{2}+\Delta & -ipk_{x} & 0\\ 0 & ipk_{x} & E+q{\left|k\right|}^{2}-\Delta & 0\\ -ipk_{x} & 0 & 0 & E-q{\left|k\right|}^{2}-\Delta \end{array}\right),$$

where E is the onsite energy, $k_{x}$ is the x‐component of the wave vector k, p is the coefficient of the linear term of $k_{x}$, q is the coefficient of the quadratic term of k, Δ is the energy detuning between the electric dipolar states and the magnetic dipolar states, respectively. When Δ=0, the accidental degeneracy of electric dipolar states and magnetic dipolar states occur at the Brillouin zone center at the frequency of semi‐Dirac point $\omega_{s}=c\sqrt{E}$, and the eigenvalues of the Hamiltonian are $E\pm \sqrt{p^{2}k_{x}^{2}+q^{2}{\left|k\right|}^{4}}$. Near the center of the Brillouin zone, the dispersions along $k_{x}$, $k_{y}$ and $k_{z}$ can be derived from the eigenvalues via a Taylor expansion:

(3) $$\omega_{k}=\left\{\begin{array}{c} \omega_{s}\pm \frac{pk_{x}c^{2}}{2\omega_{s}}\\ \omega_{s}\pm \frac{qk_{y}^{2}c^{2}}{2\omega_{s}}\\ \omega_{s}\pm \frac{qk_{z}^{2}c^{2}}{2\omega_{s}} \end{array}\right.,$$

indicating linear dispersion along $k_{x}$ and quadratic dispersions along $k_{y}$ and $k_{z}$. When the size of the mesh L changes, the electric and magnetic dipolar states are no longer degenerate, corresponding to a non‐zero Δ. Here p and q are phenomenological coefficients introduced to distinguish the linear and quadratic terms in the band dispersion near the Γ point. They serve to characterize the dispersion type associated with double‐zero or single‐zero effective parameters, which shall be demonstrated later. We choose three cases of PCs with L=0.32, L=0.327 and L=0.35, corresponding to Δ<0, Δ=0, and Δ>0, respectively. When $k_{x}=0$, Figure 2 shows their dispersion surfaces near the Γ point in the $k_{y}{-k}_{z}$ plane. As expected from the Hamiltonian, quadratic dispersions along the $k_{y}$ and $k_{z}$ directions are found when Δ=0.

**FIGURE 2:**
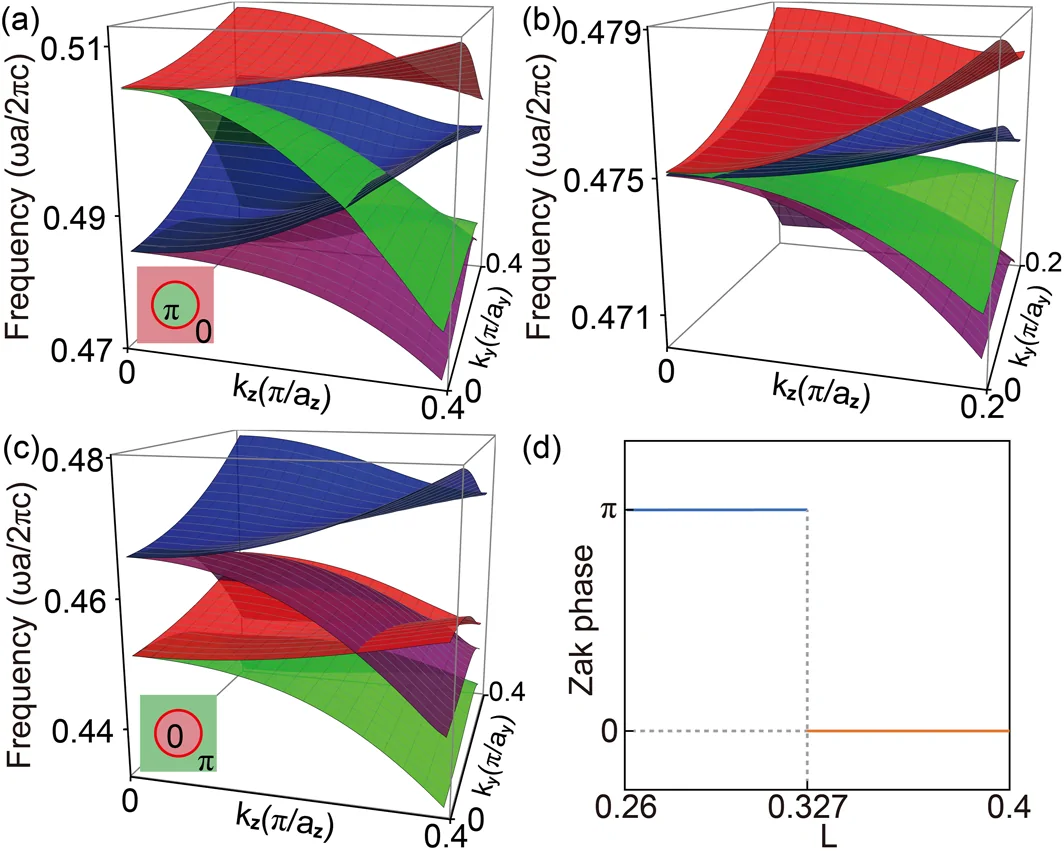
The dispersion surfaces near the Γ point in the $k_{y}{-k}_{z}$ plane when (a) L=0.32, (b) L=0.327, and (c) L=0.35. The inset graphs in (a) and (c) show the Zak phase of band 3 in and out of the nodal rings. (d) The Zak phase defined along $k_{x}$ as a function of *L* for fixed $\left(k_{y},k_{z}\right)=\left(0,0\right)$.

In contrast to 2D systems, where semi‐Dirac points often result from the accidental degeneracy of a monopolar state and a dipolar state [40], semi‐Dirac points in 3D electromagnetic systems arise from a different mechanism due to the absence of electromagnetic monopolar state. In the 3D PC, the anisotropy of the unit cell in Figure 1 split both the electric and magnetic dipolar states into doubly‐degenerate dipolar‐*y*,*z* states and a non‐degenerate dipolar‐*x* state, respectively. When Δ=0, the electric and magnetic dipolar‐*y*,*z* states form the fourfold degenerate semi‐Dirac point. As shown in Equation (3), in the momentum space, the two branches of dipolar‐*y*,*z* states always have opposite dispersions near the semi‐Dirac point. Therefore, the band dispersions inevitably cross when Δ≠0, giving rise to a nodal ring in the $k_{y}{-k}_{z}$ plane. The detailed difference between 2D semi‐Dirac point and 3D semi‐Dirac point can be found in Supporting Information S1: Note S1. For L=0.32 and L=0.35, the Zak phase are obtained by integrating the eigenstates along $k_{x}$ for every ($k_{y}{,k}_{z}$) and are plotted in the insets of Figure 2a,c (see Supporting Information S1: Note S2 for details of the calculation of Zak phase). In terms of the Zak phase, the $k_{y}{-k}_{z}$ plane can be divided into two parts, inside and outside the nodal ring, characterized by Zak phases of π and 0, respectively. The π/0 inversion of the Zak phase in the $k_{y}{-k}_{z}$ plane is induced by the band inversion of electronic and magnetic dipolar states. As illustrated in Figure 2d, for fixed $\left(k_{y},k_{z}\right)=\left(0,0\right)$, the Zak phase undergoes a transition between 0 and π at the critical point L=0.327, indicating that the fourfold semi‐Dirac point marks a topological phase transition. An interface formed by two such PCs with different Zak phases therefore supports topological interface states, as presented in Supporting Information S1: Note S2. When $k_{z}=0$, the dispersion surfaces in the $k_{x}{-k}_{y}$ plane near the Γ point are plotted in Figure 3. When L=0.327, linear dispersion along $k_{x}$ and quadratic dispersion along $k_{y}$ are observed near the semi‐Dirac point. For L=0.32 and L=0.35, the projection of the nodal ring in the $k_{x}{-k}_{y}$ plane forms a pair of Dirac points. The dispersion surfaces in Figures 2 and 3 are consistent with the dispersions described by the Hamiltonian (see Supporting Information S1: Note S3).

**FIGURE 3:**
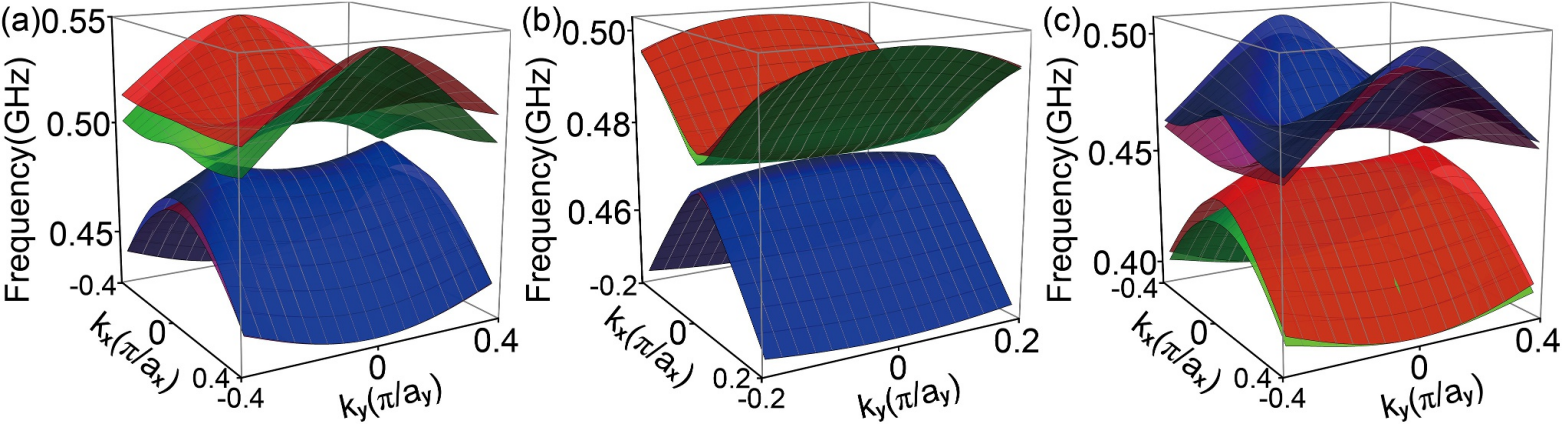
The dispersion surfaces near the Γ point in the $k_{x}{-k}_{y}$ plane when (a) L=0.32, (b) L=0.327, and (c) L=0.35.

## 3 Polarization Independent Directional Double‐Zero‐Index Property

The 3D semi‐Dirac point can be understood from an effective medium perspective. Figure 4a shows an enlarged view of the band structure near the Brillouin zone center, where the directional linear dispersion can be associated with directional double‐zero‐index effective parameters. In general, when both the effective permittivity and permeability simultaneously vanish at the same frequency, their first‐order frequency expansions lead to a dispersion relation of the form $k^{2}\propto {\left(\omega -\omega_{s}\right)}^{2}$, giving rise to linear bands. In contrast, if only one constitutive parameter crosses zero while the other remains finite, the dispersion follows $k^{2}\propto \left(\omega -\omega_{s}\right)$, resulting in quadratic bands (see Supporting Information S1: Note S4 for the details). Thus, the dispersion of our PC indicates directional double‐zero‐index property. Along the *x*‐direction, the effective parameters of the PC are obtained by an effective medium theory based on field‐averaging (see Supporting Information S1: Note S4 for the details), as illustrated in Figure 4b. Both the effective permittivity $\varepsilon_{y,z}$ and the effective permeability $\mu_{y,z}$ cross zero at the frequency of semi‐Dirac point $\omega_{s}=0.475\left(2\pi c/a\right)$, indicating that the PC can be effectively regarded as a DZIM for both $E_{z}$ and $E_{y}$ polarized electromagnetic waves along the *x*‐direction. Along the *y*‐direction, the PC behaves as a SZIM, with effective parameters $\varepsilon_{z}=0$, $\mu_{x}=0.81$ for $E_{z}$ polarized waves slightly above $\omega_{s}$ and $\varepsilon_{z}=0$, $\mu_{x}=-0.81$ slightly below $\omega_{s}$. For $E_{x}$ polarized waves, the PC has effective parameters $\varepsilon_{x}=0.29$, $\mu_{z}=0$ slightly above $\omega_{s}$ and $\varepsilon_{x}=-0.29$, $\mu_{z}=0$ slightly below $\omega_{s}$. The opposite sign of effective parameters above and below $\omega_{s}$ is a typical phenomenon when the other effective parameter cross zero [51]. Detailed information about the effective parameters can be found in the Supporting Information S1: Note S5.

**FIGURE 4:**
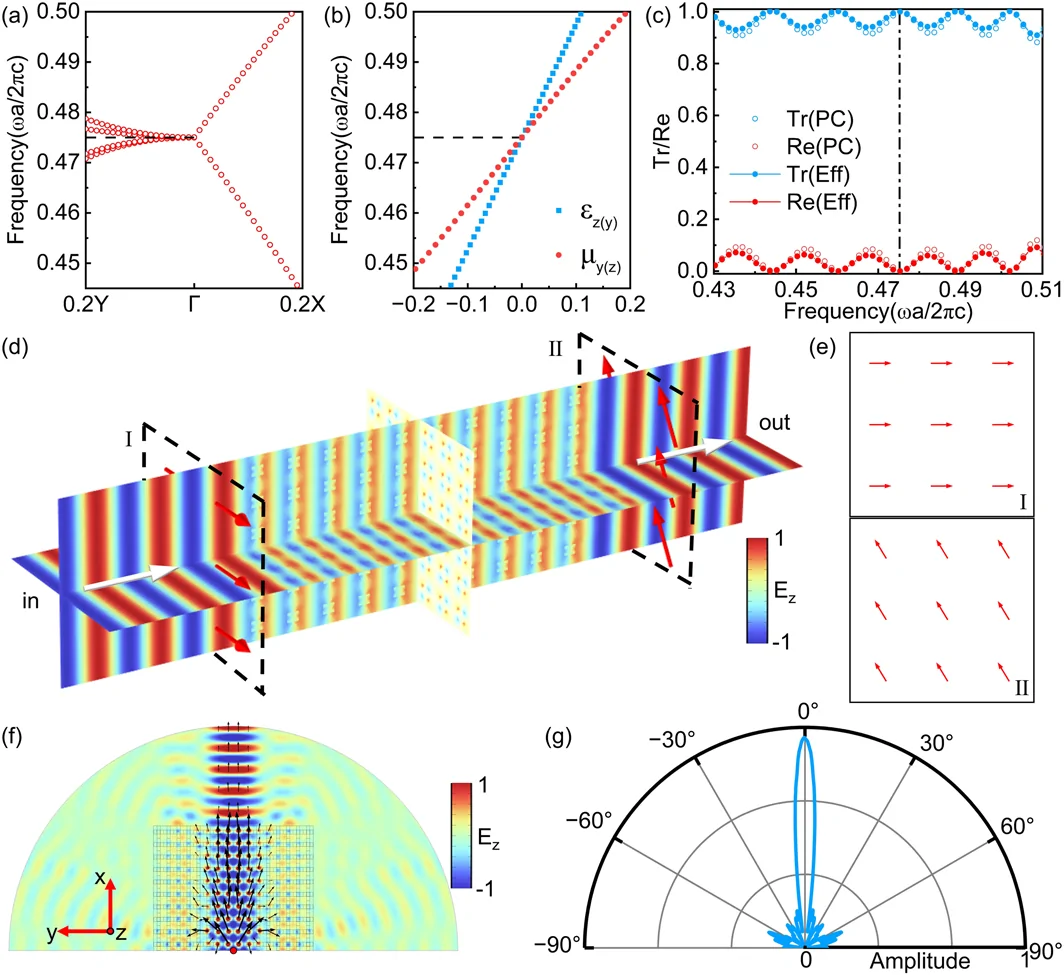
Directional polarization‐independent double‐zero‐index properties associated with the semi‐Dirac point. (a) Band structure near the semi‐Dirac point. (b) The effective permittivity $\varepsilon_{y,z}$ (blue square dots) and effective permeability $\mu_{y,z}$ (red dots) along $k_{x}$. The black dashed line corresponds to the frequency of semi‐Dirac point. (c) Transmittance and reflectance spectra for a normally incident circularly polarized electromagnetic wave to the PC consists of 10 layers of unit cell (circles) and its effective medium (dots). (d) Electric field distribution $E_{z}$ when a circularly polarized wave normally incident on the PC with frequency $\omega_{s}$. The arrows in dashed boxes represent the direction of electric field. (e) Arrow maps of electric field in the cut planes (dashed box I and II) in (d) before and after the PC. (f) The electric field distribution $E_{z}$ when an out‐of‐plane line source (red dot) with frequency $\omega_{s}$ is embedded in the PC composed of 16×10 unit cells. The black arrows denote the Poynting vectors. (g) The far‐field radiation patterns of the outgoing wave in (f).

To validate the effective parameters, the transmittance and reflectance of a circularly polarized plane wave incident on the PC and its effective medium along the *x*‐direction are presented in Figure 4c, which shows good agreement between the PC and its effective medium near $\omega_{s}$. At $\omega_{s}$, the transmission of both the PC and the effective medium reach unity due to the double‐zero‐index property for both *y*‐ and *z*‐polarized wave components along the *x*‐direction. Figure 4d shows the simulated distribution of $E_{z}$ at $\omega_{s}$, when a circularly polarized plane wave normally incident on the PC, demonstrating a total transmission. Although the electric field inside the PC is non‐uniform due to the Bloch mode, it remains continuous at the interface with the air (see Supporting Information S1: Note S7 for the details). Additionally, the arrow maps of the electric field before entering and after exiting the PC, correspond to the two dashed boxes in Figure 4d, are presented in Figure 4e, exhibiting a clear change in the polarization during propagation.

The directional double‐zero‐index property leads to a direction‐dependent response of the PC. To illustrate the directional‐selective propagation property of our PC, we place an out‐of‐plane line source at the boundary of a PC with 16×10 unit cells in *xy‐*plane and set the periodic boundaries along the *z*‐direction. As shown in Figure 4f, the simulated electric field at $\omega_{s}$ maintains nearly zero phase accumulation throughout the PC, indicating a vanishing wavevector due to the double‐zero effective parameters. As a result, the outgoing wave emerges as a highly collimated plane wave. The normalized far‐field intensity of electric field is plotted in Figure 4g as a function of the transmission angle, showing that the outgoing wave is mainly confined within a narrow range within ±5°. Such transmission behavior confirms that the electromagnetic waves can propagate with minimal angular dispersion as well as phase accumulation along the direction where the PC is effectively a DZIM, while propagation is prohibited along directions where the PC behaves as a SZIM. It can be understood from a wave filtering perspective. The line source contains components with different parallel wave vectors. Because of the directional double‐zero‐index behavior, components with large parallel wave vectors are progressively suppressed inside the PC during propagation. As a result, the transmitted wave exhibits an improved directivity. We should also emphasize that replacing the out‐of‐plane line source with a Gaussian beam does not lead to a reduction of the main‐lobe divergence, indicating that the collimation is not sensitive to the incident beam. In contrast, increasing the propagation length inside the PC significantly enhances the directivity of the transmitted wave (see Supporting Information S1: Note S8 for the details). Such characteristics indicate that our PC enables the construction of cladding‐free waveguides and beam collimation effect with low divergence after long‐distance propagations.

Here are some additional discussions about experimental feasibility and robustness of the directional DZIM. The proposed 3D PC can be realized at microwave frequencies, where PCs composed of connected dielectric meshes with ε=9 have been experimentally demonstrated [36]. Moreover, the existence of semi‐Dirac point does not rely on specific permittivity. By varying the permittivity and adjusting the geometric parameters accordingly, the semi‐Dirac point persists, suggesting that the design principle can, in principle, be extended toward higher‐frequency regimes. Numerical simulations further show that the collimation effect persists in the presence of structural defects, confirming the robustness of the proposed design against inevitable manufacturing defects (see Supporting Information S1: Note S9 for the details).

## 4 Conclusion

In this work, we propose a strategy for achieving a directional DZIM in a 3D PC. The band structure of the PC exhibits a four‐fold semi‐Dirac point at the Brillouin zone center, exhibiting linear dispersion as well as a double‐zero‐index response along $k_{x}$ and quadratic dispersions as well as single‐zero‐index response along $k_{y}$ and $k_{z}$. Unlike their 2D counterparts arising from the accidental degeneracy of monopolar states and non‐degenerate dipolar states, the semi‐Dirac point in the 3D PC marks a topological transition between two nodal rings with opposite Zak phases. As an effectively directional DZIM, the PC enables polarization‐independent perfect transmission along $k_{x}$ at the frequency of semi‐Dirac point. Because waves cannot propagate along $k_{y}$ and $k_{z}$, our PC holds strong potential for wave manipulation applications, such as polarization‐independent waveguides without the need for conventional claddings. This directional confinement mechanism opens opportunities for advanced wave manipulation in 3D photonic systems.

## Author Contributions

**Rui Dong:** software, data curation, writing – original draft. **Ying Wu:** funding acquisition, writing – review and editing. **Changqing Xu:** supervision, funding acquisition, visualization, writing – review and editing, formal analysis, investigation.

## Supporting information


Supporting Information S1

## Data Availability

Data that support the findings of this study are available in the supplementary material of this article.
