# Supplementary material for: Directional Polarization‐Independent Double‐Zero‐Index Material in a Three‐Dimensional Photonic Crystal With a Fourfold Semi‐Dirac Point
Source: Nanophotonics. 2026 Mar 20;15(6):e70045. doi: 10.1002/nap2.70045 (PMC13642564; doi:10.1002/nap2.70045)
Supplement: Supplementary file 1 [file NAP2-15-e70045-s001.docx]

**Supplementary Material of “Directional** **polarization-independent double-zero-index material in a three-dimensional photonic crystal** **with a fourfold semi-Dirac point”**

Rui Dong^1^, Ying Wu^2,3#^, Changqing Xu^1*^

^1^Key Laboratory of State Manipulation and Advanced Materials in Provincial Universities, School of Physics and Technology, Nanjing Normal University, Nanjing 210023, China

^2^Division of Computer, Electrical and Mathematical Science and Engineering, King Abdullah University of Science and Technology (KAUST), Thuwal 23955-6900, Saudi Arabia

^3^Physical Science and Engineering Division, King Abdullah University of Science and Technology (KAUST), Thuwal 23955-6900, Saudi Arabia

Emails: [ying.wu@kaust.edu.sa](mailto:ying.wu@kaust.edu.sa); changqing.xu@nnu.edu.cn

**1.	Dispersions near the semi-Dirac point in 2D and 3D PCs.**

To illustrate the distinct formation of semi-Dirac points in 2D and 3D electromagnetic systems, we present the band structures obtained from their effective Hamiltonians. Near the semi-Dirac point in 2D electromagnetic system, the tight-binding Hamiltonian can be written in the basis of a monopolar state and a non-degenerate dipolar state as follows:

$\hat{H}=\left( \begin{matrix} E+q\left| \boldsymbol{k} \right|^{2}+\Delta& -ipk_{x} \\ ipk_{x} & E-q\left| \boldsymbol{k} \right|^{2}-\Delta\end{matrix} \right)$, (S1)

where $E$ is the onsite energy, $k_{x}$ is the $x$-component of the wave vector $\boldsymbol{k}$, $p$ is the coefficient of the linear term of $k_{x}$, $q$ is the coefficient of the quadratic term of $\boldsymbol{k}$, $\Delta$ is the energy detuning between the monopolar state and the dipolar state. Figure S1(a-c) shows the evolution of a two-fold semi-Dirac point, resulting from the accidental degeneracy between the monopolar state (blue lines) and the non-degenerate dipolar state (yellow lines). This process is demonstrated by gradually increasing the frequency of the monopolar state relative to the dipolar state. In Fig. S1(a), a band gap exists before the degeneracy of these two states. When the frequency of monopolar state approaches that of the dipolar state, a twofold semi-Dirac point exists at the $\Gamma$ point as shown in Fig. S1(b), exhibiting linear dispersion along the $\Gamma X$ direction and quadratic dispersion along the $\Gamma Y$ direction. In Fig. S1(c), further increasing the frequency of monopolar state leads to the formation of a Dirac point along the $\Gamma Y$ direction.

In contrast, Fig. S1(d-f) shows the evolution of a fourfold semi-Dirac point in a 3D electromagnetic system, originating from the accidental degeneracy between doubly degenerate electric dipolar states (blue lines) and doubly degenerate magnetic dipolar states (yellow lines). By tuning the relative frequency of the magnetic dipolar states upward, Figs. S1(d) and S1(f) show the band dispersions when the electric and magnetic dipolar states intersect with each other along the $\Gamma Y$ direction, forming two nodal rings in $k_{y}{-k}_{z}$ plane as discussed in the main text. Figure S1(e) shows the accidental degeneracy of electric dipolar states and magnetic dipolar states, with a fourfold semi-Dirac point at the $\Gamma$ point, characterized by linear dispersion along the $\Gamma X$ direction and quadratic dispersions along the $\Gamma Y(\Gamma Z)$ direction.

**2.	Zak phase of the nodal ring.**

For each point in $k_{y}{-k}_{z}$ plane in our PC, the nodal ring can be regarded as a one-dimensional system along $k_{x}$. The band topology of such a system can be described by Zak phase. In $k_{y}{-k}_{z}$ plane, the Brillouin zone is divided into two regions, i.e., inside and outside the nodal ring. As shown in Fig. S2(a), one path close to the nodal ring is selected in each region. For the case of $\Delta>0$ in Hamiltonian, band 2 and band 3, which compose ring node, are shown along each path in Fig. S2(b) and Fig. S2(c), respectively. For each band, the Zak phase inside (outside) the nodal ring can be calculated by an integration along the corresponding path [46]:

$\theta=\prod_{i=1}^{N} arg(\left\langle\varphi_{i} | \varphi_{i+1} \right\rangle)$, (S2)

where the Brillouin zone along $k_{x}$ is divided into $N$ segments ($i$ ranges from 1 to $N$), $\varphi_{i}$ is the wave function of the $i_{th}$ segment. For $\Delta>0$, the Zak phase of the lower band (upper band) in Fig. S2(b) and Fig. S2(c) is $0(\pi)$ and $\pi(0)$, respectively. For $\Delta<0$, the Zak phase of the lower band (upper band) in Fig. S2(d) and Fig. S2(e) is calculated to be $\pi(0)$ and $0(\pi)$, respectively. The inversion of the Zak phase inside and outside the nodal line ring comes from the inversion of band 2 and band 3; the inversion of the Zak phase for $\Delta>0$ and $\Delta<0$ comes from the inversion of electric dipolar states and magnetic dipolar states. The semi-Dirac point in our study corresponds to the topological phase transition point.

As shown in Fig. S3(a), we consider a supercell composed of ten unit cells with $L=0.3$ and ten unit cells with $L=0.35$ stacked along the 𝑥 direction. For $k_{z}=0$, $k_{z}=0.2 \pi/a$, $k_{z}=0.4 \pi/a$ and $k_{z}=0.6 \pi/a$, the projected band structures of this photonic crystal slab along $k_{y}$ are plotted in Fig. S3(b), Fig. S3(c), Fig. S3(d) and Fig. S3(e), respectively. In all cases, interface states are clearly observed in the incomplete projected band gap, providing direct evidence of the Zak phase mismatch across the interface. For certain $k_{z}$, one branch of the interface state may overlap with the bulk spectrum. Figure S3(f) shows the electric field distributions of interface states at $\left( k_{y}, k_{z} \right)=(0.4 \pi/a,0.4 \pi/a)$.

**3.	The dispersions near the semi-Dirac point obtained from the Hamiltonian.**

When $k_{x}=0$, Figs. S4(a), S4(b) and S4(c) show the dispersion surfaces obtained from the Hamiltonian (Eq. 1 in the main text) near the $\Gamma$ point in the $k_{y}{-k}_{z}$ plane with $\Delta<0$, $\Delta=0$, and $\Delta>0$, respectively. Figures S4(d), S4(e), and S4(f) show the dispersion surfaces obtained from the Hamiltonian in the $k_{x}{-k}_{y}$ plane when $k_{z}=0$. Both of them are qualitatively consistent with the dispersion surfaces of the PC.

**4. Relation between band dispersion and effective zero-index properties.**

When a PC can be described by an effective medium under the single-mode approximation (i.e., only one Bloch eigenstate is excited by an incident plane wave), its effective constitutive parameters are strongly correlated with the band dispersion. Based on a Taylor expansion near the Brillouin zone center, the effective permittivity $\varepsilon_{eff}$ and effective permeability $\mu_{eff}$ of the PC can be expressed as

$\left\{ \begin{matrix} \varepsilon_{eff}\approx{\varepsilon(\omega_{\varepsilon})+c}_{\varepsilon}\Delta\omega_{\varepsilon}+o(\Delta\omega_{\varepsilon}) \\ \mu_{eff}\approx\mu(\omega_{\mu})+c_{\mu}\Delta\omega_{\mu}+o(\Delta\omega_{\mu}) \end{matrix} \right.$, (S3)

where $\Delta\omega_{\varepsilon}=\omega-\omega_{\varepsilon}$ and $\Delta\omega_{\mu}=\omega-\omega_{\mu}$. Here, $\varepsilon(\omega_{\varepsilon})$ and $\mu(\omega_{\mu})$ denote the zero order values of the effective parameters at frequencies $\omega_{\varepsilon}$ and $\omega_{\mu}$, respectively; $c_{\varepsilon}$ and $c_{\mu}$ are the first order expansion coefficients, and $o(\Delta\omega_{\varepsilon,\mu})$ represents higher orders.

When $\omega_{\varepsilon}=\omega_{\mu}$, i.e., the effective permittivity and permeability simultaneously vanish at the same frequency $\omega_{SD}$, the dispersion relation nearby follows

$\boldsymbol{k}^{2}\approx c_{\varepsilon}c_{\mu}\omega_{s}^{2}\Delta\omega_{s}^{2}$, (S4)

which gives rise to two linear dispersion branches near $\left| \boldsymbol{k} \right|=0$. In this case, the phase velocity diverges as

$v=\pm\frac{1}{\sqrt{c_{\varepsilon}c_{\mu}}\Delta\omega_{s}}$, (S5)

corresponding to the characteristic double-zero-index response.

In contrast, when $\omega_{\varepsilon}\neq\omega_{\mu}$, meaning that $\varepsilon_{eff}$ and $\mu_{eff}$ cross zero at different frequencies, the dispersion relations near $\omega_{\varepsilon}$ and $\omega_{\mu}$ become

$\left\{ \begin{matrix} \boldsymbol{k}^{2}\approx c_{\varepsilon}\mu(\omega_{\varepsilon})\omega_{\varepsilon}^{2}\Delta\omega_{\varepsilon} \\ \boldsymbol{k}^{2}\approx c_{\mu}\varepsilon(\omega_{\mu})\omega_{\mu}^{2}\Delta\omega_{\mu} \end{matrix} \right.$. (S6)

Both relations correspond to a single-zero-index response and lead to quadratic band dispersions. Notably, the coefficients $c_{\varepsilon}$ and $c_{\mu}$ change signs when the frequency crosses $\omega_{\varepsilon}$ and $\omega_{\mu}$, respectively, indicates that the nonvanishing effective parameter $\mu(\omega_{\varepsilon})$ and $\varepsilon(\omega_{\mu})$ naturally exhibits opposite signs above and below the corresponding single-zero-index frequency.

In our 3D PC, the fourfold semi-Dirac point exhibits linear dispersion only along the $k_{x}$ direction, while quadratic dispersions appear along the orthogonal $k_{y}$ and $k_{z}$ directions. As a result, the double-zero-index property is directional, existing only along the direction of $k_{x}$, while the $k_{y}$ and $k_{z}$ directions behave as single-zero-index media.

**5.	The calculation of effective medium parameters.**

By the effective medium theory based on field-averaging, the effective parameters of the PC along the *y*-direction can be obtained and plotted in Fig. S5. Assuming that the electromagnetic waves with frequency $\omega$ and $E_{z}$ polarization travels in the PC along $k_{x}$ direction, we can define the impedance of the PC as follows:

$Z_{x}=\frac{\left\langle E_{z} \right\rangle}{\left\langle H_{y} \right\rangle}$. (S7)

Thus, the PC can be viewed as a uniform medium with relative effective permittivity and permeability

$\varepsilon_{z}=-\frac{k_{x}}{\omega\varepsilon_{0}Z_{x}}$, $\mu_{y}=-\frac{k_{x}Z_{x}}{\omega\mu_{0}}$, (S8)

where $\left\langle\ldots\right\rangle$ represents the average of fields at the boundary of unit cell, $\varepsilon_{0}$ and $\mu_{0}$ are the permittivity and permeability of vacuum, respectively. For $E_{y}$ polarization, the PC has the impedance $Z_{x}^{'}=\frac{\left\langle E_{y} \right\rangle}{\left\langle H_{z} \right\rangle}$, and corresponding effective permittivity and permeability

$\varepsilon_{y}=\frac{k_{x}}{\omega\varepsilon_{0}Z_{x}^{'}}$, $\mu_{z}=\frac{k_{x}Z_{x}^{'}}{\omega\mu_{0}}$. (S9)

The effective parameters $\varepsilon_{y,z}$ and $\mu_{y,z}$ near the semi-Dirac point are plotted in Fig. 4(b) in the main text. Similarly, when the electromagnetic wave travels along $k_{y}$, the effective permittivity and permeability can be obtained as

$\varepsilon_{z}=\frac{k_{y}}{\omega\varepsilon_{0}Z_{y}}$, $\mu_{x}=\frac{k_{y}Z_{y}}{\omega\mu_{0}}$, $\varepsilon_{x}=-\frac{k_{y}}{\omega\varepsilon_{0}Z_{y}^{'}}$, $\mu_{z}=-\frac{k_{y}Z_{y}^{'}}{\omega\mu_{0}}$, (S10)

for $E_{z}$ polarization and $E_{y}$ polarization, respectively, where

$Z_{y}=\frac{\left\langle E_{z} \right\rangle}{\left\langle H_{x} \right\rangle}$, $Z_{y}^{'}=\frac{\left\langle E_{x} \right\rangle}{\left\langle H_{z} \right\rangle}$. (S11)

Near the frequency of semi-Dirac point, the effective parameters along $k_{y}$ are plotted in Fig. S5. The PC behaves as a SZIM, with effective parameters $\varepsilon_{z}=0$, $\mu_{x}=0.81$ for $E_{z}$ polarized waves slightly above $\omega_{s}$ and $\varepsilon_{z}=0$, $\mu_{x}=-0.81$ slightly below $\omega_{s}$. For $E_{x}$ polarized waves, the PC has effective parameters $\varepsilon_{x}=0.29$, $\mu_{z}=0$ slightly above $\omega_{s}$ and $\varepsilon_{x}=-0.29$, $\mu_{z}=0$ slightly below $\omega_{s}$.

**6.**	**Transmission properties associated with nodal ring.**

In Fig. S6, we investigate the transmission properties associated with the nodal ring, i.e., the electric and magnetic dipolar states are no longer degenerate at the center of the Brillouin zone. We choose the case of $L=0.35$ shown in Fig. 2(c) and Fig. 3(c) of the main text, corresponding to the case of $\Delta>0$ in the Hamiltonian. Figure S6(a) illustrates a plane wave polarized along *z*, i.e., $\vec{E}=(0,0,E_{z})$, at frequency $\omega=0.456(2\pi c/a)$, incident onto different surfaces of PCs with 10 layers along one direction and infinitely large in the other two directions. For example, in Fig. S6(b) we plot the transmission spectrum as a function of incident angle $\alpha$ for a PC of 10 layers thick along the *x*-direction and infinitely large along the *yz*-plane. The transmittance reaches unity only at an incident angle of $18^{\circ}$, correspond to the position of Dirac point shown in Fig. 3(c). For incident angles larger or smaller than $18^{\circ}$, the directional band gaps halt transmittance. When the incident surface is the *xz*-plane, the transmission spectrum to incident angle $\beta$ is plotted in Fig. S6(c), which shows transmittance $\sim0.59$ for normal incidence. When the incident angle increases, the transmittance decreases because of the directional band gap for nonzero $k_{x}$ in Fig. 3(c). When the incident angle tends to $30^{\circ}$, some eigenstates away from the nodal ring are excited, the transmittance becomes higher again. The transmission spectrum to incident angle $\gamma$ is plotted in Fig. S6(d). In the range of $0^{\circ}<\gamma<18^{\circ}$, the presence of the nodal ring leads to a certain degree of transmission. A sharp transmission peak exists around $\gamma=17.2^{\circ}$, corresponds to the boundary of nodal ring. When $\gamma\geq18^{\circ}$, the transmittance decreases rapidly due to the directional band gap. The transmission spectrum is consistent with the band structure.

**7.	The electric field distribution in Fig. 4(d).**

In a photonic crystal, the Bloch mode generally exhibits a strongly spatial inhomogeneity within each unit cell, so both the amplitude and the component of electric field can vary significantly depending on the position and the selected cut plane. To clarify this point, we plot the intensity of electric field $\left| E \right|$ over the entire simulation region in Fig. S7, as well as additional cross-sectional views at different locations in Fig. S8. These figures demonstrate that the observed amplitude variation in Fig. 4(d) originates from the intrinsic Bloch-mode rather than from reflection or impedance mismatch. Specifically, although the electric field distribution inside the PC is spatially non-uniform, it remains continuous at the interface with the air background and satisfies Bloch periodicity.

**8.	The effect of the incident beam and propagation length on directivity of PCs.**

To check whether the directivity is sensitive to the beam of incidence, we replace the out-of-plane line source used in Fig. 4(f) of the main text with a Gaussian beam, as shown in Fig. S9. The Gaussian beam has a waist of $2a$. Figure S9(a) shows the corresponding electric field distribution $E_{z}$. The far-field intensity of electric field in the outgoing wave is plotted in Fig. S9(b). Compared with the line source excitation, the Gaussian beam effectively suppresses the weak radiation components at large angles, while the angular divergence of the main radiation lobe remains nearly $\pm5^{\circ}$. This result indicates that the far-field divergence is not sensitive to the incident beam. Instead, it is governed by the band dispersion of the photonic crystal.

As shown in Fig. S10, the propagation length inside the photonic crystal plays a crucial role in determining the divergence of the outgoing wave. Figure S10(a) shows the far-field radiation pattern for a photonic crystal composed of 10 layers of unit cells along the propagation direction, excited by an out-of-plane line source. When the number of layers is increased to 20 and 30, the beam becomes progressively more collimated, as demonstrated in Fig. S10(b) and Fig. S10(c), respectively. The electric field distribution $E_{z}$ for the case of 30 layers is shown in Fig. S10(d).

**9.	The discussions about experimental feasibility and robustness of the directional DZIM.**

The material with relative permittivity $\varepsilon=9$ employed in our work is available in aluminum oxide at microwave frequencies, and connected dielectric meshes have already been experimentally realized. Recent works, Ref [36], have demonstrated that such structures can be fabricated by assembling photonic crystal slabs with identical cross sections into blocks. These established fabrication strategies provide a realistic and practical route for implementing our proposed design in the microwave regime. Moreover, the underlying physical mechanism of the fourfold semi-Dirac point does not rely on a specific permittivity. Figure S11 illustrates the band structure of the photonic crystal when the permittivity and geometric parameter of dielectric meshes are set as $\varepsilon=5$ and $L=0.376$, respectively. When the dielectric permittivity is reduced from $\varepsilon=9$ to $\varepsilon=5$, which is achievable using materials in the visible spectrum such as Si_3_N_4_ or TiO_2_, although the geometric parameter changes, the existence of semi-Dirac point remains robust as the critical point separating two nodal-ring phases with opposite Zak phases. The relaxation of material requirements suggests that the design principle can, in principle, be extended toward higher-frequency regimes.

To address the influence of inevitable manufacturing defects, we introduce two kinds of defects in the photonic crystal shown in Fig. 4(f) of the manuscript. In Fig. S12(a), we introduce two specific unit cells within the photonic crystal with geometric parameter $L=0.36$. In Fig. S12(b), we coat the surfaces of dielectrics with a metallic film in two unit cells. When an out-of-plane line source at the frequency $\omega_{s}=0.475({2\pi c}/{a)}$ is embedded in the photonic crystal, the simulated electric field distributions are plotted in Fig. S12(c) and Fig. S12(d), the normalized far-field radiation patterns are plotted in Fig. S12(e) and Fig. S12(f), respectively. For both cases, the outgoing wave is still mainly confined along the *x* direction, demonstrating that the collimation effect remains functional under defects.

Fig. S1. The scheme for semi-Dirac points in 2D and 3D electromagnetic systems. The band structures are obtained from their effective Hamiltonians. (a-c) Band structures (a) before, (b) at, and (c) after the degeneracy between a monopolar state (blue line) and a non-degenerate dipolar mode (yellow line) in 2D momentum space, show (a) a band gap, (b) a twofold semi-Dirac point at the $\Gamma$ point, and (c) a Dirac point along the $\Gamma Y$ direction, respectively. (d-f) Band structures (d) before, (e) at, and (f) after the degeneracy between twofold electric dipolar states (blue lines) and twofold magnetic dipolar states (yellow lines) in 3D momentum space, show (d, f) the nodal rings discussed in the main text and (e) a fourfold semi-Dirac point at the $\Gamma$ point.

Fig. S2. The calculation of Zak phase. (a) Brillouin zone and the integration path inside and outside the nodal ring (red). (b, c) The Zak phase of band 2 and band 3 along the (b) blue path and (c) green path when $\Delta>0$. (d, e) The Zak phase of band 2 and band 3 along the (d) blue path and (e) green path when $\Delta<0$.

Fig. S3. Topological interface states induced by the transition of Zak phase. (a) Schematic of the supercell composed of 10 unit cells with $L=0.3$ (green) and 10 unit cells with $L=0.35$ (blue) stacked along the 𝑥 direction. (b-e) Projected band structures of the photonic crystal slab along $k_{y}$ for (b) $k_{z}=0$, (c) $k_{z}=0.2 \pi/a$, (d) $k_{z}=0.4 \pi/a$ and (e) $k_{z}=0.6 \pi/a$. In all cases, interface states appear in the projected bulk band gap, arising from the mismatch of the Zak phase across the interface.

Since the two states cannot always be distinguished, they are both represented by red dots in the figures. (f) The electric field distributions of the interface states at $\left( k_{y}, k_{z} \right)=(0.4 \pi/a,0.4 \pi/a)$.

Fig. S4. The dispersion surfaces near the $\Gamma$ point obtained from the Hamiltonian for (a) $\Delta<0$, (b) $\Delta=0$, and (c) $\Delta>0$ in the $k_{x}{-k}_{y}$ plane, (d) $\Delta<0$, (e) $\Delta=0$, and (f) $\Delta>0$ in the $k_{y}{-k}_{z}$ plane.

Fig. S5. The effective parameters along $k_{y}$ near the semi-Dirac point: the effective permittivity $\varepsilon_{z}$ (blue square dots) and effective permeability $\mu_{x}$ (yellow rhombus dots) are for $E_{z}$ polarized electromagnetic waves; the effective permittivity $\varepsilon_{x}$ (green triangle dots) and effective permeability $\mu_{z}$ (red dots) are for $E_{x}$ polarized electromagnetic waves.

Fig. S6. The transmission properties associated with the nodal ring. (a) The schematic of the PC the different direction of incidence. The geometric parameter of the meshes is set as $L=0.35$. The transmittance varies with incident angle (b) *α*，(c) *β* and (d) *γ* when incident waves come from different directions.

Fig. S7. The intensity of electric field in the whole simulation region when a circularly polarized wave normally incident on the PC.

Fig. S8. Cross-sectional views of the electric field intensity at different locations.

Fig. S9. The transmission behavior when the line source in Fig. 4(f) is replaced by a Gaussian beam. (a) The electric field distribution $E_{z}$. (b) The far-ﬁeld radiation patterns of the outgoing wave in (a).

Fig. S10. Far-field radiation patterns of the outgoing wave for photonic crystals with different propagation lengths. (a-c) Far-field radiation patterns with (a) 10, (b) 20, and (c) 30 layers of unit cells along the propagation direction, with divergence angles (a) $\pm5^{\circ}$, (b) $\pm3.1^{\circ}$, and (c) $\pm2.4^{\circ}$, respectively. (d) Electric field distribution $E_{z}$ for the case of 30-layered structure. The red dot represents the out-of-plane line source.

Fig. S11. Band structure of the photonic crystal when the permittivity and geometric parameter of dielectric meshes are set as $\varepsilon=5$ and $L=0.376$, respectively. The fourfold semi-Dirac point (solid circle) still exist at the $\Gamma$ point.

Fig. S12. The robustness of wave collimation in the photonic crystal. (a) Illustration of enlarge two unit cells within the photonic crystal with geometric parameter $L=0.36$. (b) Illustration of coat the surfaces of dielectrics with a metallic film in two unit cells. (c, d) Simulated electric field distributions radiated by an out-of-plane line source located at the red dots, respectively. (e, f) Simulated far-field radiation patterns of the outgoing wave in (c) and (d), respectively.
